# Is Working Memory Necessary for Both Selection and Calculation in Level 1 Visual Perspective Taking? Evidence from Children and Adults

**DOI:** 10.3390/bs14100897

**Published:** 2024-10-03

**Authors:** Guifen He, Wei Song, Peiqi Dong, Canmei Xu, Yanlin Zhou, Haoqin Ye, Qiong Zhang

**Affiliations:** 1 Department of Psychology and Behavioral Sciences, Zijingang Campus, Zhejiang University, Hangzhou 310058, China; hgf520@zju.edu.cn (G.H.); 21739040@zju.edu.cn (W.S.); peiqi.dong@zju.edu.cn (P.D.); 11739004@zju.edu.cn (Y.Z.); 2 Department of Parenting and Special Education, Faculty of Psychology and Educational Sciences, KU Leuven, 3000 Leuven, Belgium; canmei.xu@kuleuven.be; 3 Department of Preschool Education, Lishui University, Lishui 323400, China; yehaoqin@lsu.edu.cn

**Keywords:** visual perspective taking, perspective calculation, perspective selection, verbal working memory, spatial working memory, children

## Abstract

Previous research has suggested that there are two modular processes of perspective selection and calculation in level 1 perspective taking. Evidence further showed that verbal working memory is associated with both processes in adults. However, research has not tested whether verbal working memory may be associated with working memory in children. Moreover, since perspective taking is associated with spatial working memory, it is necessary to investigate whether spatial working memory links to both processes. By recruiting 9-year-old children and college students in the single-task paradigm and the dual-task paradigm, we conducted two experiments to answer these questions. Results in experiment 1 suggested that verbal working memory correlated with adults’ perspective calculation, but with both processes in children’s perspective taking. Results in experiment 2 showed that spatial working memory is associated with adults’ perspective calculation and children’s perspective selection. These findings suggest that different components of working memory play distinct roles in the processing of perspective taking, which is moderated by age.

## 1. Introduction

Theory of mind (TOM) refers to the ability or process of reasoning with others’ thoughts, beliefs, knowledge, and feelings and to predict behavioral responses [1]. Embedded in the framework of ToM, visual perspective taking (VPT) refers to the ability to know and understand what someone else might see when an object is simultaneously visible to both the self and the other person [2].

Researchers have been interested in the cognitive basis of ToM [3]. Leslie and colleagues proposed that there are two distinct processes that contribute to ToM, one is calculation, which refers to the processing of what someone sees, knows, or thinks. The other is selection, which points to the processing of making judgment [4,5]. Based on empirical evidence, Leslie and colleagues claimed that ToM calculation is fast, automatic, and cognitively efficient, while selection of ToM is resource demanding, requiring effortful mobilization of executive function. Thus, researchers posited that VPT includes the same two cognitive processing stages as well.

A dot judgment task is commonly used in the investigation of cognitive processes of visual perspective taking. In this task, participants would see an avatar standing before a wall, on both sides of which a set of dots are displayed. They are asked to judge either the number of dots that they themselves could see (self-perspective) or the number of dots that the avatar could see (other-perspective). If the avatar faces the wall and all the dots are on one side of the wall, the avatar could see all the dots; this makes a consistent trial. Otherwise, if the avatar faces one side of the wall but dots are on both sides, the avatar will see fewer than the participant, resulting in an inconsistent trial. Performance in inconsistent conditions was compared for both self and other judgments. In the other-perspective condition, participants were slower and more error prone when their own perspective differed from the avatar’s, because of the ‘egocentric’ effect. Similarly, an ‘altercentric’ effect was observed in the self-perspective condition, which was described as the influence of other agents’ presence on our own information processing [6] and demonstrated by slower and more error-prone responses when the avatar’s perspective was in conflict with the participant’s self-perspective.

Furthermore, a dual-task paradigm was used to tease apart calculation and selection processes and the role of general executive resources in these two sub-processes as well [7]. The basis logic is that by taxing the secondary task, there will be differences in processing costs between the single and dual conditions for the inconsistent trials. If the calculation process is affected on self-perspective trials, the altercentric interference effect as compared to the effect observed in the single-task condition would be reduced. In contrast, the egocentric interference effect would remain or increase in dual-task conditions compared with when the task is performed alone. If the selection process is affected, both in consistent trials and inconsistent trials, the egocentric and altercentric interference effects would increase in the dual-task condition compared with when the task is performed alone [7]. The first trial conducted by Qureshi et al. [7] used a dual-task and found that a dual-task (in which participants were required to do incongruent key press, i.e., one to two tones, two to one tone [8]) assessing inhibitory control did not affect the calculation process, but did affect the selection process. Then, Qureshi and Monk [9] used another task which serves to mobilize working memory, and they found that executive function links with both perspective calculation and perspective selection. These results suggested that a parsimonious depiction of which sub-processes of perspective taking are cognitively demanding is warranted.

Similar to Qureshi and Monk [9], the present study is interested in the role of working memory on visual perspective taking. Working memory refers to a limited capacity system that temporarily holds and manipulates information [10]. Working memory has been argued to be one of the most fundamental functions underlying higher cognitive functions [11,12]. According to two commonly applied models, working memory (WM) is a composite ability involving different components. Firstly, in Baddeley’s model [13], the phonological loop, the visuospatial sketchpad, the episodic buffer, and the central executive comprise the four fundamental components, among which the phonological loop represents storage of auditory–verbal information and a rehearsal mechanism, the visuospatial sketchpad provides temporary storage and rehearsal of visual and spatial information. Similarly, in Miyake and colleagues’ theory [14], WM is sustained by two distinctive pools of domain-specific resources when processing verbal and visuospatial information.

Accordingly, the present study aimed to explore whether the two working memory sub-components play a role on the perspective taking processes. First, we used a backward digit span task to measure verbal working memory (experiment 1). This task requires additional executive control resources and can be described as a measure of working memory, especially in children [15,16]. Then, we used a forward Corsi span task to measure spatial working memory (experiment 2). When taking different perspectives, participants need to not only compute how many numbers an avatar can see but also compute the location of objects, that is, they need to deal with both visual and spatial information [7,9,17,18]. Thus, we proposed the notion that visuospatial working memory is associated with perspective taking. To note, both tasks were used in a dual-task paradigm to investigate the role of either verbal working memory or visuospatial working memory on perspective taking processes.

Concerning the relation of working memory and perspective taking, previous research found that children’s perspective taking ability improves with age and this ability is related to age-related increases in working memory capacity; it means that before they reach maturation, both abilities and their relation might be different from those for adults [19]. However, other studies suggested that even adults still show a strong bias towards their own perspective as children, especially when reasoning about what someone else knows or thinks [3,20]. That is, there might be some similarity in children and adults. To deal with the controversy, the present study aimed to recruit adults to replicate and verify the previous findings on adults at the first point, and secondly, to examine whether this influence could extend to children. To note, we were not interested in the age difference. Instead, we aimed to explore the distinct roles of either verbal working memory or spatial working memory on the sub-processes of perspective taking for children and adults.

Based on the previous literature, we hypothesized that verbal working memory and spatial working memory would play different roles in the sub-processes of perspective taking for either children or adults. It remains unclear how each component of working memory specifically affects perspective processes.

## 2. Experiment 1

### 2.1. Participants

Based on the results of our pilot studies, we predicted a medium effect size (Cohen’s *f* = 0.20) for our experimental design [21]. A power analysis using G*power 3.1 determined that given an α level of 0.05 and a power of 0.95, the sample size required to achieve the predicted effect size was approximately 52 individuals for each group. The sample for children is overpowered, it also allows us to establish evidence of any effect using Bayesian factor (BF) hypothesis testing.

Two groups of participants were recruited. One group of 63 undergraduates participated for course credit. One was excluded from the final analysis of missing data in either task. Thus, the final sample for this group was 62, including 45 females (mean age = 21.76 years; SD = 2.46). Another group of 99 typically developing children were recruited from a primary school in an eastern city of China. Three of them were excluded from the final analysis of missing data in either task. The final sample for children was 96, including 58 girls (mean age = 114.6 months; SD = 0.62).

All of the children were from upper-middle-income families. All participants were in good health, with normal or corrected-to-normal vision. A written consent form was obtained from parents and teachers for all of the children, and written informed consent was obtained from all undergraduates before the experiments. The study was approved by the ethical committee of our university.

### 2.2. Procedure Perspective Taking

Participants first completed the verbal working memory task alone, and then their maximum verbal working memory span minus one was used for the dual-task condition. The participants then practiced the task in both the single and dual-task conditions, performing ten trials in each condition. We used the same procedure and trials of the adult version as that of Qureshi [9], and in order to ensure the children can successfully complete the task, the children’s version had been modified and simplified, which are described below for each task.

### 2.3. Measures

All participants were asked to report their handedness before they performed the tasks. They were then invited to complete a verbal working memory task, a single perspective taking task, and then, and a perspective taking dual task. All tasks were programmed in E-prime2.0 (Psychological Software Tools—PST, Inc., Pittsburgh, PA, USA). Children were tested on a laptop with a resolution of 1024 × 768 px and a 14 in TFT (thin-film-transistor) display in a one-to-one setting. All the tasks are described below.

#### 2.3.1. Perspective Taking Single-Task

The visual perspective taking task used the same stimuli and procedure of Samson et al. [17] for adults. To make the task more child friendly, we used a longer duration for children. We also had pilot testing for specific presentation time and number items. Each trial began with a fixation cross presented in the center of the screen for 500 ms. In both groups, a cue “You see X” or “He/She see X” was presented for 750 ms (adult) or 1000 ms (child). “You” or “He/She” indicated whose perspective to respond from. “X” indicated a number between 0 and 3 for undergraduates and 0 and 2 for children. Then, the participant would see a room with red dots on the left and right walls. A human avatar stood in the center of the room facing left or right. Participants were asked to verify whether the number of dots visible from the cued perspective was the same with the given number or not by pressing “Q” on the keyboard for “yes” and “P” for “no. If no response was given within 3000 ms (adult) or 5000 ms (child), the next trial would be prompted. For both groups, there were ten practice trials (five consistent trials and five inconsistent trials), but one hundred four test trials (fifty-two trials × two blocks) in total for adults and thirty-two test trials (sixteen trials × two blocks) for children. An additional practice block was administered if participants’ response accuracy percentage did not reach 80%. Accuracy and reaction time were recorded.

#### 2.3.2. Backward Digit Span Task (BDST)

The task used a similar task and procedure to that of Prencipe et al. [1] and Alloway [22]. In this task, participants were asked to listen to a set of numbers and then were asked to repeat those numbers in the reverse order. The sequence length increased from three to eight, with three trials each length. If a participant made at least two errors within a certain length, the task was terminated. The dependent variable was the maximum length of the correct sequence. The task was the same for adults and children.

#### 2.3.3. Perspective Taking Dual-Task

**Figure 1:**
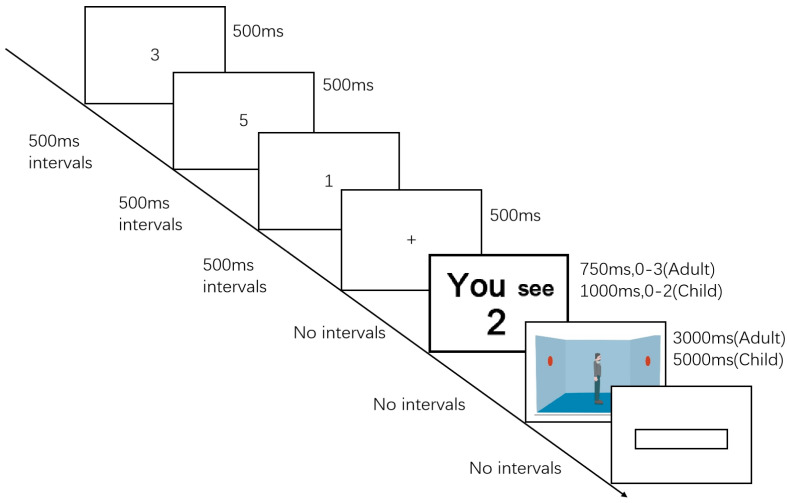
Example of different processes in the perspective taking dual–task (Verber working memory). Note: The number of dots visible from each perspective was either the same (consistent) or different (inconsistent). Participants verified if a given number (0–3) matched the number of dots visible either from their own perspective (“YOU”) or from the avatar’s perspective (“HE”).

The task used the stimuli and procedure of Qureshi et al. [9]. In the dual-task condition, participants were required to finish both a BDST and a perspective task at the same time. First, they were asked to listen to a sequence of digits, they had to remember the order of the digits and repeat them in a reverse order after they finished a trial of a perspective taking task. The maximum sequence length of BDST was calculated by the maximum of single BDST subtracted from one. The presentation time and the number of the cue were different between the adults and the children (see Figure 1). For adults, there were ten practice trials (five consistent trials and five inconsistent trials) and one hundred four test trials (fifty-two trials × two blocks) in total. For children, there are only five practice trials and sixty-four test trials (thirty-two trials × two blocks). An additional practice block was administered if participants’ response accuracy percentage did not reach 80%. Nine children needed extra practice blocks, but no adults required extra practice. Accuracy and reaction time were recorded (see Figure 1).

### 2.4. Data Analysis

First, we analyzed error rates for each participant by condition in the visual perspective taking task. With reference to Samson’s [18] analysis method, the trials of verbal working memory task were correct, and the answers with “Q” were analyzed in the results. Prior data screening removed any response times either less than 250 ms or more than 3 SD from the mean, by condition. Omissions resulting from participants timing out were also removed. The exclusion rates for adults and children were 1.04% and 1.20%, respectively. Then, a repeated measures analysis of variance (ANOVA) was conducted using consistency (consistent, inconsistent), perspective (self, other), and task condition (single, dual) as independent variables.

Then, to make sure whether WM is associated with either the automatic process of calculating process or the control process of selection in the perspective taking task, we performed process-dissociation procedure (PDP) analysis for each participant [7]. Specifically, to understand altercentric intrusion effects, an automatic process (A) and a controlled process (C) are computed. In a consistent trial, calculating the avatar’s perspective automatically (A) and explicitly responding with one’s own perspective explicitly (C) results in the probability of responding correctly being the sum of C and A when C fails: P = C + A (1 − C) in the case of correct consistent trials. During inconsistent trials, the probability of an incorrect response is calculated by the probability of the automated process (A) operating when the controlled process (C) is fails: P = A (1 − C) in the case of incorrect inconsistent trials. Solving these two equations algebraically yields separate estimates for C and A:

C = P (correct|consistent trials) − P (incorrect|inconsistent trials)

A = P (incorrect|inconsistent trials)/(1 − C)

In a dual-task paradigm, the performance was usually compared with that in a single-task. That is, if the calculation process is affected on self-perspective trials, the altercentric interference effect in a dual task would reduce. In contrast, the egocentric interference effect would remain or increase in dual-task conditions. If the selection process is affected, the egocentric and altercentric interference effects would increase in the dual-task condition. These predictions are summarized in Table 1.

### 2.5. Results

#### 2.5.1. Verbal Working Memory

The verbal working memory capacity of children ranged from 2 to 6, with an average of 3.58 ± 1.07. Adults’ verbal working memory capacity was nearly 6.98 ± 1.84, ranging from 4 to 9.

#### 2.5.2. Error Rates

**Figure 2:**
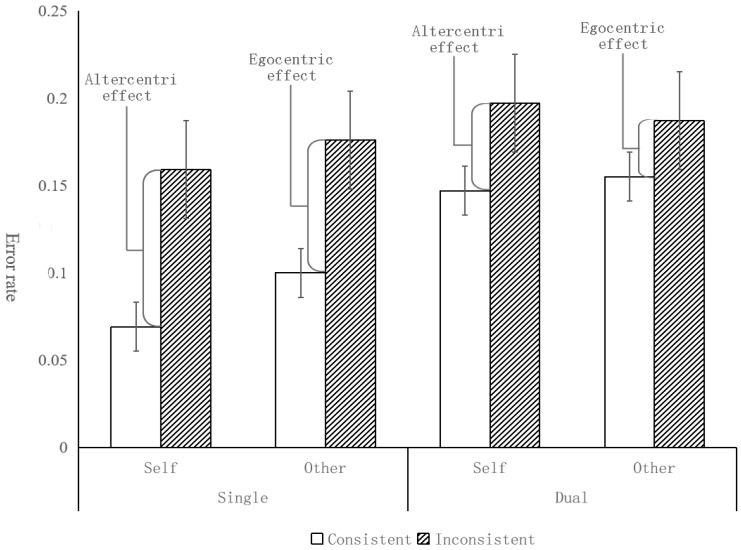
Error rates by task condition, consistency, and perspective for children (error bar = 95% confidence intervals).

In the children group, the results are depicted in Table 2 and Figure 2. A significant main effect of task condition was found [*F*(1, 95) = 8.31, *p* < 0.01, *η_p_*^2^ = 0.08; BF_10_ = 13.99], the error rate (12.7%) in single-task was significantly lower than that of the dual-task condition (17.1%), suggesting that children made more wrong judgments in the dual-task condition than in the single-condition. A significant main effect of consistency [*F*(1, 95) = 15.42, *p* < 0.001, *η_p_*^2^ = 0.14; BF_10_ = 1200.65] was found; the error rate (11.8%) in the consistent condition was significantly lower than that in the inconsistent condition (18%), suggesting that children made more errors in the inconsistent condition, which means that they are prone to take the avatar’s perspective when making a judgment. We failed to find a significant main effect of perspective [*F*(1, 95) = 0.67, *p* = 0.42, *η_p_*^2^ = 0.007; BF_10_ = 0.11], showing that children performed similarly when taking both self and other’s perspective. No significant interaction effect was found either between task condition and perspective [*F*(1, 95) = 1.17, *p* = 0.28, *η_p_*^2^ = 0.012; BF_10_ = 0.19], between task condition and consistency [*F*(1, 95) = 2.74, *p* = 0.10, *η_p_*^2^ = 0.028; BF_10_ = 0.31], between perspective and consistency [*F*(1, 95) = 0.35, *p* = 0.56, *η_p_*^2^ = 0.004; BF_10_ = 0.14], or among task condition, perspective, and consistency [*F*(1, 95) = 0.00, *p* = 0.98, *η_p_*^2^ = 0.000; BF_10_ = 0.12]. As can be seen from Figure 2, compared with single-task conditions, the difference between inconsistent and consistent trials has a decreasing trend (but not significant), indicating the altercentric interference effect and the egocentric interference effect was reduced but was not significant. These results suggested that the verbal working memory task had no significant effect on the calculation process of children’s perspective taking.

**Figure 3:**
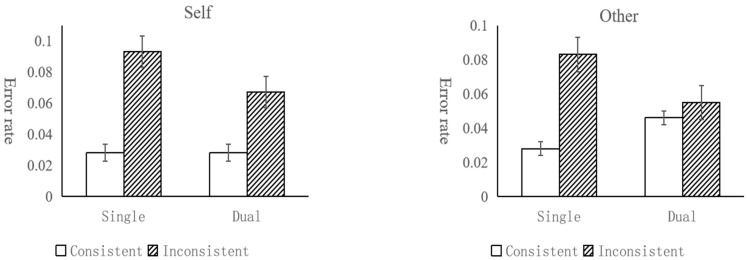
Error rates by task condition, consistency, and perspective in adult group (error bar = 95% confidence intervals).

For the adult group, the results are depicted in Figure 3, we found a significant main effect of consistency [*F*(1, 62) = 50.55, *p* < 0.001, *η_p_*^2^ = 0.45; BF_10_ = 9.32× 10^8^], with significantly higher error rates (7.4%) on inconsistent trials than consistent trials (3.2%). That is, adults would automatically calculate the avatar’s perspective whether the experiment requires them or not. No significant main effect of perspective was found [*F*(1, 62) = 0.06, *p* = 0.81, *η_p_*^2^ = 0.001; BF_10_ = 0.10], suggesting that adults performed as well when taking other people’s perspective as taking self’s perspective. There was no main effect of Task condition [*F*(1, 62) = 1.74, *p* =0.19, *η_p_*^2^ = 0.027; BF_10_ = 0.31], indicating that adults had similar error rate in both the single-task condition and the dual-task condition. A significant interaction effect between task condition and consistency was found [*F*(1, 62) = 9.27, *p* < 0.01, *η_p_*^2^ = 0.13; BF_10_ = 12.96], simple effect analysis showed that the significant difference between consistent and inconsistent trials was larger in the dual-condition than in the single-condition (*p* < 0.01, Cohen’s *D* = 0.38; BF_10_ = 8.80). That is, both the altercentric interference effect and egocentric interference effect are reduced under the dual-task condition. The results show that verbal working memory has an effect on the processing of adult perspective taking, and the calculation of this effect has a more significant effect, but the influence on the selection process cannot be excluded. The interaction between perspective and consistency [*F*(1, 62) = 3.01, *p* = 0.09, *η_p_*^2^ = 0.046; BF10 = 0.50] was not significant. No significant effect was found either between task condition and perspective [*F*(1, 62)= 0.46, *p =* 0.50, *η_p_*^2^ = 0.007; BF_10_ = 0.17] or among task condition, perspective, and consistency [*F*(1, 62) = 0.88, *p* = 0.35, *η_p_*^2^ = 0.014; BF_10_ = 0.26].

#### 2.5.3. PDP Analyses

Using equations described earlier, we computed estimates of A (automatically calculating the avatar’s perspective) and C (explicitly responding with one’s own perspective) for each group of participants.

For the children’s perspective taking performance, C was significantly weaker in the dual-task condition (M = 0.66, SD = 0.27) than in the single-task condition (M = 0.75, SD = 0.24) (*t*(95) = 2.88, *p* = 0.005, Cohen’s *D* = 0.294; BF_10_ = 5.37). Furthermore, A was also significantly weaker in the dual-task condition than in the single-task condition (dual: M = 0.59, SD = 0.35; single: M = 0.68, SD = 0.36) (*t*(95) = 2.00, *p* = 0.048, Cohen’s *D* = 0.205; BF_10_ = 0.77). Concerning the ANOVA’ s result, no significant interaction effect between task conditions and consistency was elicited, making it hard to discern whether verbal working memory has an effect on either of the two perspective-taking sub-processes. Only after combining with the results of PDP analyses could we conclude that verbal working memory was linked with both the automatic and controlled processes.

For the adults’ perspective taking, the analysis showed that C was stronger in the dual-task condition (M = 0.91, SD = 0.09) than in the single-condition (M = 0.88, SD = 0.11) (*t*(61) = 2.76, *p* = 0.008, Cohen’s *D* = 0.348; BF_10_ = 4.36). However, A was also significantly weaker in the dual-task condition than in single-task condition (dual: M = 0.71, SD = 0.37, alone: M = 0.84, SD = 0.20) (*t*(61) = 2.59, *p* = 0.012, Cohen’s *D* = 0.327; BF_10_ = 2.98). The dual task of verbal working memory enhanced the selection process of adults’ perspective taking slightly but had a greater negative effect on the calculation process, which aligns with the ANOVA’s analysis. This suggested that, for adults, the influence of verbal working memory on the calculation process is more prominent.

### 2.6. Discussion

In the present study, we employed a VPT task in conjunction with a verbal working memory to test the effect of verbal working memory on the process of visual perspective taking. We found that spontaneously effects of taking self and other’s perspective not only in adults but also in children. Furthermore, verbal working memory affected the calculation of perspective taking for adults, but it had influence on both the selection and calculation processes for children.

As for the results in adults, the calculation process is impaired in the dual-task condition, while the selection process shows a slight upward trend, suggesting that verbal working memory will participate in both the calculation and selection perspectives but has a more significant influence on the calculation process. This is a partial replication of previous finding, with which both selection and calculation processes were impaired in the dual-task condition [9]. This inconsistency might be due to the different stimuli used in these two studies, that is, forward letter span task in the study of Qureshi et al. [9] and backforward number span in our study. In backward digit span task, the transposition of digit’s order requires the involvement of executive attentional resources [23]. Thus, it performs more as a working memory task [16]. That is, it requires more resources than forward digit span task [24]. However, the reason for why a resource-demanding dual-task only affects the calculation process awaits further investigation.

As for children, we found that they exhibited egocentric intrusion larger than the interference from others, which was consistent with previous findings [25]. This suggested that individuals show egocentric interference from as early as childhood. However, their ability to take on the perspective of others increased with age, which helps individuals adapt to society [26,27]. Moreover, this result gives a hint that taking other people’s perspectives might depend on one’s own perspective, which in turn helps individuals develop and adjust their own perspectives [27].

Furthermore, we found that the mobilization of working memory resources leads to a significant decline in both children’s perspective calculation and selection processes, suggesting that children’s verbal working memory is involved in both the calculation and selection processes of perspective taking. That is, for children, working memory develops along with perspective taking ability, but it is possible that before reaching maturation, the relation between working memory and perspective taking might be different from those for adults. Another possibility that does not exclude the previous one could be that working memory, being one of the core components of executive function, develops gradually from as early as 3 years old, differentiates at the age of 4, and is not completely separated until the age of 15 [28,29]. That is, in young children, inhibition control and cognitive flexibility, the other two core components of executive function, have not fully differentiated. This means that the development of working memory overlaps with that of the other two components. Therefore, when the working memory resources were occupied, both the selection and calculation processes were impaired. On the contrary, in relatively fully developed adults, core executive functions, such as working memory, inhibitory control, and cognitive flexibility, were more fully differentiated. Therefore, the calculation process was more significantly affected by working memory, while the selection process was more affected by inhibitory control, another crucial component of executive function [7].

Moreover, as we aimed to explore how the two sub-components of working memory play roles in the perspective taking processes, experiment 2 will further explore the role of spatial working memory on visual perspective taking. To note, the same paradigm and a classical task tapping spatial working memory will be employed.

## 3. Experiment 2

### 3.1. Participants

Ninety participants consisting of third and fourth graders from an urban primary school in Zhejiang Province as were recruited as the subjects of the children group (43 girls; mean age = 113.5 months; SD = 0.58), and fifty-five undergraduate students (42 girls; mean age = 21.86 years, SD = 2.47) from a university in a city of Zhejiang Province were recruited as adult group subjects. Parental permission for child participation was obtained before data collection. The study was approved by the ethical committee at Zhejiang University.

### 3.2. Design

**Figure 4:**
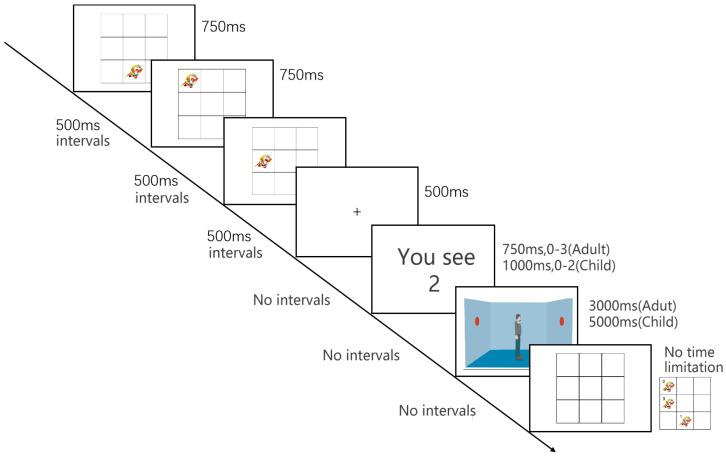
Example of different processes in the dual–task perspective taking. (Visuospatial working memory).

We used the exact same procedure as in experiment 1, except that the secondary task in the dual-task condition was spatial working memory. This study aimed to explore the effect of spatial working memory on visual perspective taking in children and adults. (See Figure 4).

#### Forward Corsi Span Task

The task used the similar task and procedure of Corsi [30] and Kessels et al. [31]. This task was used to tap visuospatial WM. In this task, participants were presented with 3 × 3 square grids on the computer monitor, and the Monkey King would appear in the grids randomly at any-one-location once per trial. After the presentation, participants were asked to click each of the grids in forward order by tapping the blank grids on the screen. Two trials were given at each list length, with list lengths increasing by one as children moved through the test. The test terminated when the participant failed to remember the sequence for two consecutive trials at any level. The dependent variable was the maximum set size that children achieved.

### 3.3. Results

#### 3.3.1. Spatial Working Memory

The spatial working memory capacity of children ranged from 2 to 6, with an average of 4.02 ± 0.86. Adults’ spatial working memory capacity was significantly larger than that of children, reaching nearly 6.81 ± 1.61 and ranging from 4 to 9.

#### 3.3.2. Error Rates

**Figure 5:**
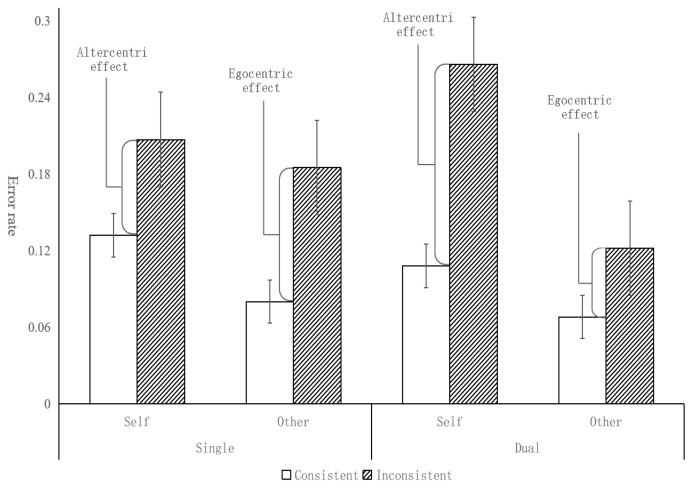
Error rates by task condition, consistency, and perspective in the children group (error bar = 95% confidence intervals).

In the child group, the results are depicted in Figure 5, the main effect of perspective was significant [*F*(1, 89) = 14.85, *p* < 0.001, *η_p_*^2^ = 0.143; BF_10_ = 1661.71], the error rate (17.8%) was significantly higher with the children’s own perspective than that (11.3%) of the avatar’s. There was also a significant main effect of consistency [*F*(1, 89) = 34.61, *p* < 0.001, *η_p_*^2^ = 0.28; BF_10_ = 4.25 × 10^5^], with a higher error rate (19.5%) on inconsistent trials than that (9.7%) on consistent trials. The main effect of task condition was not significant [*F*(1, 89) = 0.29, *p* = 0.59, *η_p_*^2^ = 0.003; BF_10_ = 0.10]. There was significant two-way interaction between task condition and perspective [*F*(1, 89) = 4.16, *p* < 0.05, *η_p_*^2^ = 0.045; BF_10_ = 0.48], and simple effect analysis found that the error rate in the self-perspective was significantly higher than that in the other-perspective (*p* = 0.02, Cohen’s *D* = 0.25; BF_10_ = 1.55) in the dual-task condition. The results showed that the error rate in the dual-task condition was higher than that of the single-task for self-perspective, while the error rate in the dual-task condition was significantly lower than that of the single-task for others’ perspective. We speculated that children automatically choose others’ perspective when they complete the task in self-perspective, and this process requires cognitive resources. There was no significant interaction either between task condition and consistency [*F*(1, 89) = 0.22, *p* = 0.64, *η_p_*^2^ = 0.002; BF_10_ = 0.17], or between perspective and consistency [*F*(1, 89) = 1.45, *p* = 0.23, *η_p_*^2^ = 0.016; BF_10_ = 0.21]. There was a significant three-way interaction between task condition, perspective, and consistency [*F*(1, 89) = 5.81, *p* < 0.05, *η_p_*^2^ = 0.061; BF_10_ = 2.00], and simple effect analysis found that the error rate of inconsistent trials (26.60%) in the self-perspective was significantly higher than that in the other- perspective (12.20%) (*p* < 0.001, Cohen’s *D* = 0.38; BF_10_ = 40.82) in the dual-task condition. These suggested that the selection process was impaired while the calculation process was unaffected.

**Figure 6:**
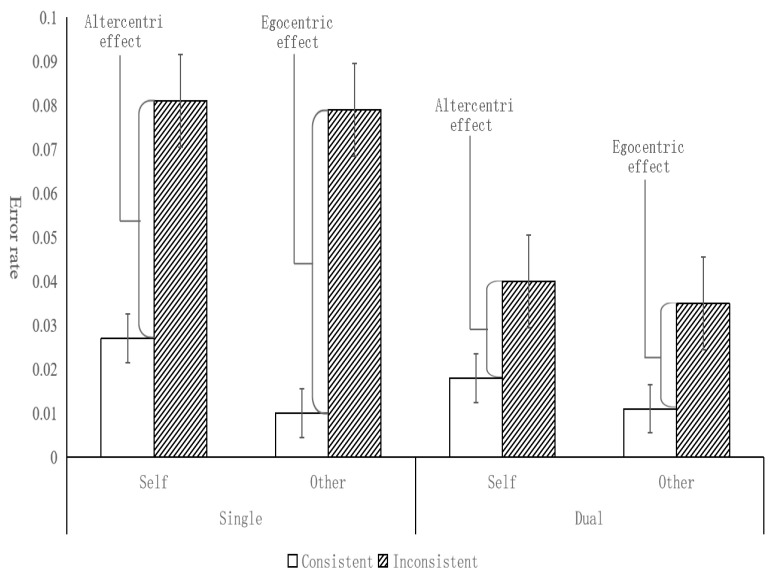
Error rates by task condition, consistency, and perspective in the adult group (error bar = 95% confidence intervals).

In the adult group, the results are depicted in Figure 6, the main effect of perspective was not significant [*F*(1, 57) = 3.81, *p* = 0.06, *η_p_*^2^ = 0.063; BF_10_ = 0.26], showing that adults performed as well when taking other people’s perspective as taking self’s perspective. The main effect of task condition was significant [*F*(1, 57) = 11.55, *p* < 0.01, *η_p_*^2^ = 0.168; BF_10_ = 619.34]. The error rate in the single-task (4.9%) was significantly higher than that in dual-task (2.6%). The main effect of consistency was significant [*F*(1, 57) = 60.31, *p* < 0.001, *η_p_*^2^ = 0.514; BF_10_ = 1.43 × 10^13^], and the error rate in the consistent condition was significantly lower (1.7%) than that under the inconsistent condition (5.9%). It suggested that adults spontaneously process the avatar’s perspective regardless of the experimental requirements. The interaction between task condition and consistency was significant [*F*(1, 57) = 10.72, *p* < 0.01, *η_p_*^2^ = 0.158; BF_10_ = 77.00], simple effect analysis showed that the difference between inconsistent trials and consistent trials was significantly larger in completing single-task than that in dual-task (*p* < 0.01, Cohen’s *D* = 0.43; BF_10_ = 15.98). The results showed that both egocentric and altercentric interference tended to decrease in the dual-task condition, indicating that the perspective calculation process was impaired. The interaction between task condition and perspective was not significant [*F*(1, 57) = 0.16, *p* = 0.69, *η_p_*^2^ = 0.003; BF_10_ = 0.16].There was no significant interaction between perspective and consistency (*F*(1, 57) = 0.91, *p* = 0.35, *η_p_*^2^ = 0.016; BF_10_ = 0.39). The interaction of task condition, perspective, and consistency was not significant either [*F*(1, 57) = 0.27, *p* = 0.60, *η_p_*^2^ = 0.005; BF_10_ = 0.20].

#### 3.3.3. PDP Analyses

Similar to experiment 1, PDP was applied to analyze the C and A, and then the paired-sample t-test was used to compare the differences between the single-condition and dual-condition. For the children’s perspective taking, C was not different between the dual-task (*M* = 0.72, *SD* = 0.28) and single-task conditions (M = 0.71, SD = 0.29) [*t*(89)= 0.33, *p* = 0.744, Cohen’s *D* = 0.034; BF_10_ = 0.12]. The same pattern was shown for A (dual-task: *M* = 0.68, *SD* = 0.41; single: *M* = 0.64, *SD* = 0.38) [*t*(89) = 0.67, *p* = 0.50, Cohen’s *D* = 0.071; BF_10_ = 0.15]. The results showed that spatial working memory has no significant effect on either selection or calculation for children. Combined with the ANOVA results, the spatial working memory has a greater influence on the perspective selection process only under the condition of self-perspective.

For the adults’ perspective taking, the analyses showed that C was stronger in the spatial working memory dual-task condition (*M* = 0.95, *SD* = 0.05) than in the alone condition (*M* = 0.90, *SD* = 0.10) [*t*(57) = 3.27, *p* = 0.002, Cohen’s *D* = 0.429; BF_10_ = 15.60], and A was weaker in the dual-task than in the single-task condition (dual-task: *M* = 0.80, *SD* = 0.33, single-task: *M* = 0.90, *SD* = 0.17) [*t*(57) = 2.12, *p* = 0.038, Cohen’s *D* = 0.279; BF_10_ = 1.15]. These results indicated that the spatial working memory task enhanced perspective selection in adults but had a negative effect on the perspective calculation. Combined with the results of the ANOVA, it can be found that spatial working memory has an influence on both perspective calculation and selection, but it had a more negative influence on the calculation process.

### 3.4. Discussion

In this experiment, spatial working memory had a more significant effect on children’s perspective selection process than the calculation process. This adds to the notion that perspective calculation is a cognitively efficient process that makes relatively few demands on EF and so is not disrupted by concurrent performance of an executive task [7]. However, in adults, spatial working memory had a more significant effect on their perspective calculation process than the selection process; this is different from the previous findings and what we found in experiment 1.

Both children and adults demonstrated relatively automatic calculation of the avatar’s perspective, which aligns with the findings in experiment 1. Moreover, both groups demonstrated lower error rates on the avatar’s perspective taking, suggesting that the visual spatial working memory task facilitates the performance of the perspective taking task [32,33].

## 4. General Discussion

This research is the first of its kind to examine the potential contribution of both the verbal working memory and visuospatial working memory to perspective calculation and selection. When performing a perspective taking task, working memory provides a buffer where representations of a stimulus need to be transformed, especially when there are various interferences [27,34]. The results we found in adults are somewhat contrasting with previous research that has suggested that inhibitory control (or attention) is important for perspective selection, whereas calculation is automatic, independent of EF [7,35] and unaffected by time demands [36]. We found that both verbal working memory and spatial working memory affected the calculation of perspective taking for adults. Our findings confirmed that although cognitive resources are particularly important for perspective selection, working memory is also imperative for calculation in adults. Our results are different from what Qureshi et al. [9] reported. That is, by using a forward digit span task, they found that both calculation and selection of adults’ perspective taking were affected by verbal working memory. The possible reason might be that the forward digit span task measures attention, which is necessary for immediate recall of information from short-term memory, while the backward digit span task we used might deploy both attention and WM abilities in information processing [37].

While for children, verbal working memory correlated with children’s calculation and selection processes, spatial working memory is associated with children’s perspective selection. It may be postulated that verbal working memory contributes to the calculation and selection of avatar perspective, while spatial working memory is a prerequisite of selection, or it facilitates this process. It also suggested that the roles of executive resources in children perspective taking are dependent on the sub-components of working memory.

In both experiments, all the children and adults showed spontaneous processing of either their own perspective or other’s perspective. This is consistent with previous findings [6,8]. These suggested that children as young as nine have already demonstrated a spontaneously perspective taking skill. Furthermore, both children and adults showed similar trend in significantly greater egocentric interference when compared to the altercentric interference effect, which echoes with previous reports [25]. That is, the process of own’s own perspective is more automated and cognitively effortless, and also, this ability emerges early since childhood and persists throughout to adulthood [26,27].

Overall, our work provided a precise picture of the effect of different working memory components on the perspective taking processes. This is important not only for advances in theory but also for identifying catered interventions for different ages. For instance, it may be that both training strategy processes are optimally effective only for children.

## 5. Limitations and Further Work

There are several limitations in the present research. First, our population consisted of typically developing children from predominantly privileged backgrounds, and, thus, our results might have been affected by restricted range issues. Second, as executive function differentiates after childhood, investigating the influence of inhibitory control and cognitive flexibility on perspective taking might provide us with a full picture of the link between executive function and perspective taking. Third, it is worth noting that our cross-sectional data might bias the effects. Although our finding aligns with the study conducted in adults that working memory exerts an influence on perspective taking processes, a longitudinal design is needed to verify the changes with this effect from childhood to adults [38]. In addition, we acknowledge that since all the participants were presented with a WM task, followed by a single VPT task and then a dual task, there might be an order effect. Overall, future studies with more representative samples that include students with wider ranges, a broader set of executive function measures, a longitudinal design, and a balanced order would bolster the generalizability of our results.

## Figures and Tables

**Table 1:** Summary of predictions of effects of a dual-task performance (working memory task) on self-perspective and other-perspective judgements if working memory is necessary for either process of calculation or selection.

	Role of Working Memory	
	Calculation	Selection
Self judgements	Decreased altercentric interference	Increased altercentric interference
Other judgements	Increased processing cost for all other judgement	Increased egocentric interference

**Table 2:** The description of children’s error rates by task condition, consistency, and perspective.

			Self-Perspective		Other-Perspective	
			M	SD	M	SD
Error rates	Single	Consistency	6.90	1.20	10.20	1.40
		Inconsistent	15.90	3.00	17.60	2.00
	Dual	Consistency	14.70	1.90	15.50	1.90
		Inconsistent	19.70	2.80	18.70	2.20

## Data Availability

The data that support the outcomes of this study are accessible from the corresponding author. Data are available from the authors with the permission of Zhejiang University. Written informed consent has been obtained from the patients to publish this paper.
